# Metoprolol compared to carvedilol deteriorates insulin-stimulated endothelial function in patients with type 2 diabetes - a randomized study

**DOI:** 10.1186/1475-2840-9-21

**Published:** 2010-05-25

**Authors:** Britt Kveiborg, Thomas S Hermann, Atheline Major-Pedersen, Buris Christiansen, Christian Rask-Madsen, Jakob Raunsø, Lars Køber, Christian Torp-Pedersen, Helena Dominguez

**Affiliations:** 1 Department of Medicine, Naestved Hospital, Naestved, Denmark; 2 Department of Cardiology, Gentofte Hospital, Hellerup, Denmark; 3 Joslin Diabetes Center, Boston (MA), USA; 4 Department of Cardiology, Rigshospitalet Heart Center, Copenhagen, Denmark; 5 Department of Cardiology, Herlev Hospital, Herlev, Denmark; 6 Department of Internal Medicine, Gentofte Hospital, Hellerup, Denmark

## Abstract

**Aim:**

Studies of beta blockade in patients with type 2 diabetes have shown inferiority of metoprolol treatment compared to carvedilol on indices of insulin resistance. The aim of this study was to examine the effect of metoprolol versus carvedilol on endothelial function and insulin-stimulated endothelial function in patients with type 2 diabetes.

**Method:**

24 patients with type 2 diabetes were randomized to receive either 200 mg metoprolol succinate or 50 mg carvedilol daily. Endothelium-dependent vasodilation was assessed by using venous occlusion plethysmography with increasing doses of intra-arterial infusions of the agonist serotonin. Insulin-stimulated endothelial function was assessed after co-infusion of insulin for sixty minutes. Vaso-reactivity studies were done before and after the two-month treatment period.

**Results:**

Insulin-stimulated endothelial function was deteriorated after treatment with metoprolol, the percentage change in forearm blood-flow was 60.19% ± 17.89 (at the highest serotonin dosages) before treatment and -33.80% ± 23.38 after treatment (p = 0.007). Treatment with carvedilol did not change insulin-stimulated endothelial function. Endothelium-dependent vasodilation without insulin was not changed in either of the two treatment groups.

**Conclusion:**

This study shows that vascular insulin sensitivity was preserved during treatment with carvedilol while blunted during treatment with metoprolol in patients with type 2 diabetes.

**Trial registration:**

Current Controlled Trials NCT00497003

## Introduction

Type 2 diabetes is associated with a high risk of cardiovascular complications [1]. Beta-blockers are generally considered to worsen metabolic control in patients with diabetes, but the GEMINI (The Glycemic Effects in Diabetes Mellitus: Carvedilol-Metoprolol Comparison in Hypertensives) study demonstrated improved metabolic control in patients with type 2 diabetes and hypertension treated with carvedilol as compared with metoprolol [2]. Further, in the presence of heart failure, carvedilol was shown to be associated with improved survival (The Carvedilol or Metoprolol European Trial [COMET]) and with fewer cases of new onset diabetes compared to metoprolol tartrate [3,4].

These results lead us to hypothesize that carvedilol and metoprolol might have different vascular effects related to insulin sensitivity. Endothelial dysfunction is thought to be one of the earliest changes in the pathogenesis of atherosclerosis [5], and is associated with an increased risk of cardiovascular disease [6]. Diabetes and insulin resistance are associated with endothelial dysfunction [7] as well as reduced insulin sensitivity of the endothelium - reduced insulin-stimulated endothelial function [8,9]. Further, improved regulation of glucose control with insulin in patients with type 2 diabetes is known to be associated with fewer vascular complications [UKPDS - (UK Prospective Diabetes Study)] [1] and we and others have shown it to result in improved endothelial insulin sensitivity [8,9] and improved endothelial function [10].

In this study, we hypothesized that the beneficial effects of carvedilol compared to metoprolol could be related to an improvement of endothelial function and/or endothelial insulin resistance in patients with type 2 diabetes.

## Methods

### Study group

A total of 19 patients with type 2 diabetes and 10 lean healthy controls were included and completed the study. Measurements of endothelial function as well as insulin stimulated endothelial function were performed in all individuals. All patients with type 2 diabetes met the diagnostic criteria for type 2 diabetes, as defined by the American Diabetes Association (ADA) [11]. None of the patients were treated with insulin. Patients with a history of atherosclerosis or heart disease of any cause were excluded, as were patients with known diabetic retinopathy, nephropathy or neuropathy. Additional exclusion criteria were known intolerance to beta-blocker treatment, bradycardia, hypotension and untreated hypertension. Patients with severe asthma or patients who received treatment with beta-agonists were also excluded from the study. None of the persons in the control group received any kind of medication and had no history of cardiovascular disease. Diabetes, hypertension and smoking were also exclusion criteria in the healthy control group.

Patients were recruited by advertisement in a local newspaper, and all patients gave written informed consent before entering the study. The study was approved by the ethics committee of the city of Copenhagen (ref KF 02-071/03), as well as the Danish Medicines Agency (ref 2612-2423).

### Design

Patients with type 2 diabetes were randomized to receive treatment with metoprolol succinate (N = 10; SeloZok, AstraZeneca, Cheshire, England) or carvedilol (N = 9; Dimitone, Roche, Basel, Switzerland). The target dose was 200 mg once daily for metoprolol and 25 mg twice daily for carvedilol in order to secure equipotent doses of the two beta-blockers. The study was designed as an open parallel group study. Before and after the two-month treatment period, endothelial function and insulin-stimulated endothelial function were measured.

#### Venous occlusion plethysmography

Forearm blood-flow was measured by using venous occlusion plethysmography as described previously [8]. All measurements were done blinded to the treatment protocol. The patients did not take their usual medication in the morning on the day of examination. All examinations were done after an overnight fast and abstinence from smoking. The patients lay supine in a quiet room, with the temperature kept constant. Both forearms were placed at a horizontal level with the right atrium while measurements were done.

An arterial cannula with an external diameter of 1 mm was inserted into the brachial artery, preferentially in the non-dominant arm. The arterial cannula was used for intra-arterial infusions and blood pressure measurement.

To assess endothelium-dependent vasodilation, forearm blood-flow was measured during infusion of increasing doses of serotonin (7, 21, 70 ng/min) [Serotonin (Clinalfa, Läufelfingen, Switzerland)]. For each dose, serotonin was infused for 4 minutes, before blood-flow measurements were done, to obtain a steady state. Measurements of forearm blood-flow were done simultaneously in both the infused and the non-infused arm, and presented as the ratio between the two arms. Endothelium-independent vasodilation in the forearm was examined by exchanging serotonin infusion with increasing doses of sodium nitroprusside [Nitropress (Abbott Laboratories, North Chicago, IL)]. The doses of sodium nitroprusside (0.5, 1 and 1.5 μg/min) were chosen according to previous studies, to ensure matching blood-flow to the flows obtained by studies of serotonin in healthy people [12,13].

Insulin-stimulated endothelial function was assessed by an intra-arterial co-infusion of serotonin and insulin. Insulin [Actrapid (Novo Nordisk Scandinavia, Malmö, Sweden) in a 1% human albumin solution (vehicle)] was infused at a rate of 0.05 mU/kg body weight/min for 60 minutes and followed by co-infusion of serotonin to achieve a dose-response study as described above. To determine the NO-dependent fraction of insulin-stimulated serotonin response, an intra-arterial co-infusion of L-NMMA [L-NMMA (Clinalfa)] was infused for 10 min, with a dose of 3.3 mg/min, followed by a dose-response study with serotonin.

To allow wash-out between measurements, all infusions were stopped for at least 30 minutes while saline was infused at a rate of 60 ml/h to maintain the cannula patent.

All blood-flow measurements are presented as a relative blood-flow given as the actual flow (ml/min) of the infused arm as a proportion to the non-infused arm. This has been done to correct for the systemic changes in flow during the day and changes in between the two days of examinations, irrespective of the infusions of substances.

### Statistics

This study is a small size study with the purpose of finding differences between both serotonin stimulated endothelial function as well as insulin stimulated endothelial function in groups. Endothelial function is presented as a mean of flow. Means of flow were compared with a paired t-test at baseline before treatment and again after treatment with either of the two beta blockers. Dose-response curves were compared by combined analysis of variance and covariance (mixed model analysis). Experimental subject and the interaction between experimental subject and dose of serotonin were entered as random variables whereas study group (metoprolol or carvedilol) dose of serotonin were entered as fixed terms. Calculations were performed with the SAS (Statistical Analysis Systems) version 9.1

## Results

Characteristics of the two treatment groups as well as the healthy control group are shown in Table 1. No significant differences were seen between the two diabetes groups at baseline. Compared to the healthy control group, the type 2 diabetic patients had a higher BMI and higher fasting glucose as well as glycosylated hemoglobin, as expected. There were no significant differences in total cholesterol between the groups, but the type 2 diabetes group tended to have higher triglycerides- and well as LDL-levels. The actual p-values for the changes between the groups are presented in Table 1.

**Table 1:** 

	T2DM Carvedilol (N = 9)	T2DM Metoprolol (N = 10)	Healthy controls (N = 10)	P Carvedilol vs. metoprolol	P Carvedilol vs. control	P Metoprolol vs. control
Age (years)	58.9 ± 2.67	58.0 ± 3.02	47.6 ± 1.89	0.83	0.003	0.009
Sex (M/F)	7/2	9/1	5/5			
Smoking (%)	1 (11)	2 (20)	0			
Oral hypoglycaemic* (%)	8 (89)	9 (90)	0			
Aspirin (%)	0	0	0			
Statins (%)	3 (33)	5 (50)	0			
ACE inhibitors/AT2 B (%)	4 (44)	3 (30)	0			
Body weight (kg)	89.72 ± 7.10	97.9 ± 5.76	75.62 ± 4.24	0.38	0.10	0.006
BMI (Kg/m²)	29.26 ± 1.76	32.22 ± 1.84	24.4 ± 0.93	0.27	0.02	0.002
Systolic BP (mm Hg)	142.4 ± 5.09	143.4 ± 5.12		0.90		
Diastolic BP (mm Hg)	71.4 ± 2.59	70.5 ± 4.67		0.87		
Heart rate (beats/min)	67.1 ± 2.16	69.4 ± 3.31		0.58		
Fasting glucose (mmol/L)	8.76 ± 0.78	8.54 ± 1.11	5.2 ± 0.17			
Fasting insulin (μU/l)	10.20 ± 3.20	10.82 ± 2.14				
Hb A1c (%)	7.6 ± 0.59	7.29 ± 0.42	5.24 ± 0.10	0.67	0.0004	0.0002
Total Cholesterol (mmol/L)	4.12 ± 0.28	3.93 ± 0.25	4.5 ± 0.29	0.62	0.36	0.15
LDL (mmol/L)	2.29 ± 0.31	2.25 ± 0.27	2.70 ± 0.26	0.93	0.29	0.22
HDL (mmol/L)	1.19 ± 0.06	1.09 ± 0.10	1.40 ± 0.14	0.42	0.19	0.09
TG (mmol/L)	1.41 ± 0.27	1.33 ± 0.26	0.90 ± 0.17	0.83	0.12	0.18
CRP (mmol/L)	3.00 ± 0.44	6.80 ± 2.38	2.00 ± 0.58	0.15	0.10	0.05

* Oral hypoglycaemic: Included patients taking both metformin and sulfonylurea

Abbreviations:T2DM: Patients with Type 2 Diabetes; M/F: Male/Female; ACE inhibitors: Angiotensin Converting Enzyme inhibitors; AT2B: Angiotensin 2 Blockers; BMI: Body Mass Index, BP: Blood Pressure; Hb A1c: Glycosylated Hemoglobin A1c; LDL: Low Density Lipoprotein; HDL: High Density Lipoprotein; TG: TriGlyceride; CRP: C-Reactive Protein.

In the group of patients treated with carvedilol or metoprolol, 3 patients were smokers whereas there were no smokers in the control group. The patients in the control group were slightly younger than the patients in the two groups with type 2 diabetes.

Twenty four patients were randomized in the study. Of those, five patients were withdrawn from the study: One because of technical difficulties in reading the results of the study and two because of difficulties with the arterial cannula. Two patients were withdrawn from the study due to adverse reactions during the treatment period: One patient who developed a severe cutaneous allergic reaction during treatment with carvedilol and one patient who had a minor stroke during treatment period with metoprolol. Two additional patients with adverse reactions during the treatment were not withdrawn from the study and results of their vaso-reactivity studies were included. Of these, one patient developed diarrhea during treatment with carvedilol and one patient in the metoprolol group had a mild episode of depression.

Ten patients with type 2 diabetes received treatment with metoprolol succinate, mean daily dose of 175 mg, and 9 patients received treatment with carvedilol, mean daily dose of 44 mg for a period of 2 months. Doses of metoprolol succinate and carvedilol were equivalent in this study which corresponds with a dose of 88% of target dose for both drugs.

Changes in baseline characteristics after treatment with either carvedilol or metoprolol are presented in Table 2 and 3 respectively. After treatment with metoprolol, there was a significant mean increase in body weight of 1.8 kg (1.8% change) (p = 0.02), whereas a non-significant mean increase in body weight of 0.6 kg (0.6% change) was seen in the carvedilol group (p = 0.43).

**Table 2:** Changes in baseline characteristics seen after treatment with carvedilol.

	Before carvedilol (N = 9)	After carvedilol (N = 9)	% Change after treatment	P-value
Body weight (kg)	89.72 ± 7.10	90.32 ± 7.39	0.6%	0.43
BMI (Kg/m²)	29.26 ± 1.76	29.48 ± 1.87	o.8%	0.39
Systolic BP (mm Hg)	142.4 ± 5.09	136.9 ± 5.80	-3.7%	0.38
Diastolic BP (mm Hg)	71.4 ± 2.59	61.3 ± 3.13³	-14.1%	0.003
Heart rate (beats/min)	67.1 ± 2.16	60.7 ± 1.74³	-9.5%	0.02
Fasting glucose (mmol/L)	8.61 ± 0.78	9.66 ± 1.31	12.2%	0.20
Fasting insulin (μU/L)	10.20 ± 3.20	13.75 ± 8.42	34.8%	0.68
Hb A1c (%)	7.6 ± 0.59	7.66 ± 0.76	0.8%	0.95
Total Cholesterol (mmol/L)	4.12 ± 0.28	4.48 ± 0.35	8.7%	0.06
LDL (mmol/L)	2.29 ± 0.31	2.5 ± 0.30	9.2%	0.10
HDL (mmol/L)	1.19 ± 0.06	1.24 ± 0.09	4.2%	0.37
TG (mmol/L)	1.41 ± 0.27	1.57 ± 0.28	11.3%	0.38
CRP (mmol/L)	3.00 ± 0.44	2.89 ± 0.26	-3.7%	0.68

T2DM: Patients with Type 2 Diabetes; M/F: Male/Female; ACE inhibitors: Angiotensin Converting Enzyme inhibitors; AT2B: Angiotensin 2 Blockers; BMI: Body Mass Index, BP: Blood Pressure; Hb A1c: Glycosylated Hemoglobin A1c; LDL: Low Density Lipoprotein; HDL: High Density Lipoprotein; TG: TriGlyceride; CRP: C-Reactive Protein.

**Table 3:** Changes in baseline characteristics seen after treatment with metoprolol.

	Before metoprolol (N = 10)	After metoprolol (N = 10)	% Change after treatment	P-value
Body weight (kg)	97.9 ± 5.76	99.7 ± 5.88	1.8%	0.02
BMI (Kg/m²)	32.22 ± 1.84	32.84 ± 1.95	1.9%	0.03
Systolic BP (mm Hg)	143.4 ± 5.12	142.8 ± 5.56	-0.4%	0.90
Diastolic BP (mm Hg)	70.5 ± 4.67	64 ± 3.84	-9.2%	0.04
Heart rate (beats/min)	69.4 ± 3.31	65.5 ± 6.74	-5.6%	0.01
Fasting glucose (mmol/L)	8.53 ± 0.99	8.67 ± 1.14	1.6%	0.90
Fasting insulin (μU/L)	10.82 ± 2.14	14.83 ± 4.75	37.1%	0.60
Hb A1c (%)	7.29 ± 0.42	7.51 ± 0.62	3.0%	0.77
Total Cholesterol (mmol/L)	3.93 ± 0.25	3.81 ± 0.27	-3.1%	0.59
LDL (mmol/L)	2.25 ± 0.27	1.99 ± 0.32	-11.6%	0.17
HDL (mmol/L)	1.09 ± 0.10	1.06 ± 0.12	-2.8%	0.10
TG (mmol/L)	1.33 ± 0.26	1.71 ± 0.35	28.6%	0.04
CRP (mmol/L)	6.80 ± 2.38	4.56 ± 1.56	-32.9%	0.19

T2DM: Patients with Type 2 Diabetes; M/F: Male/Female; ACE inhibitors: Angiotensin Converting Enzyme inhibitors; AT2B: Angiotensin 2 Blockers; BMI: Body Mass Index, BP: Blood Pressure; Hb A1c: Glycosylated Hemoglobin A1c; LDL: Low Density Lipoprotein; HDL: High Density Lipoprotein; TG: TriGlyceride; CRP: C-Reactive Protein.

Fasting blood glucose, fasting insulin and glycosylated hemoglobin remained unchanged at the end of the treatment with any of the two beta-blockers (Table 2 and 3).

Systolic blood pressure did not change with metoprolol while carvedilol treatment tended to lower systolic blood pressure although this change was non-significant (142.4 ± 5.09 mmHg before treatment versus 136.9 ± 5.80 mmHg after treatment) (-3.7% change) (p = 0.38). Diastolic blood pressure decreased after treatment with a beta-blocker to a similar level in both treatment groups; from 71.4 ± 2.59 mmHg to 61.3 ± 3.13 mmHg (-14.1% change) in the carvedilol group (p < 0.05) and 70.5 ± 4.67 mmHg to 64.0 ± 3.84 mmHg (-9.2% change) in the metoprolol group (p < 0.05), while resting heart rate decreased only significantly in the carvedilol group from 67.1 ± 2.16 to 60.7 ± 1.74 (-9.5% change) (p < 0.05).

### Endothelial function

**Figure 1:**
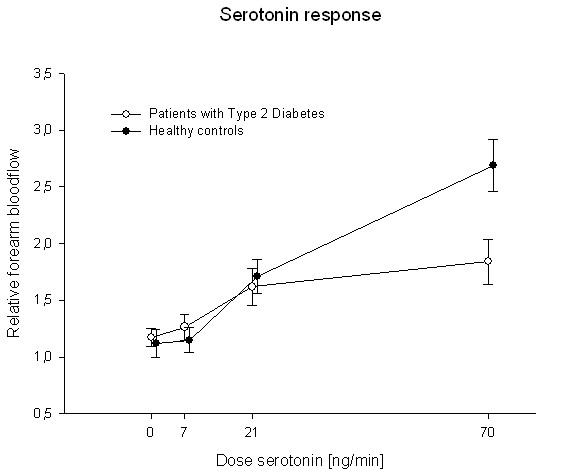
**Endothelial function presented as relative forearm blood-flow at baseline was lower in the group of patients with type 2 diabetes, compared to the healthy control group**. Forearm blood-flow is presented as a proportion between the infused and the non-infused arm.

**Figure 2:**
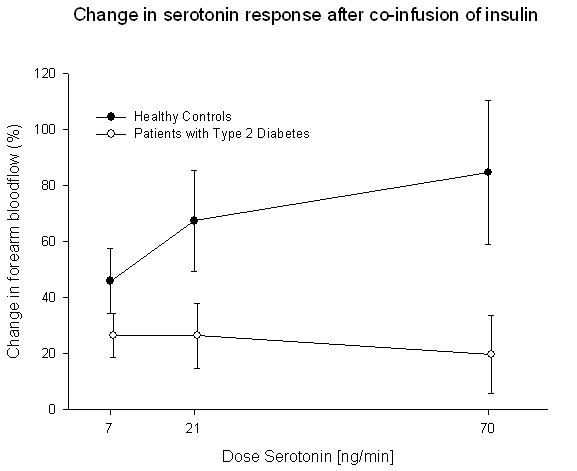
**At baseline, serotonin-stimulated forearm blood flow was enhanced by insulin in the healthy control group while this response was blunted in patients with type 2 diabetes**.

The patients with type 2 diabetes had a significant lower response to serotonin than the healthy control group (Fig. 1). Relative flow (±SEM) at baseline at the three dose levels of serotonin infusion were 1.17 ± 0.08; 1.26 ± 0.11; 1.62 ± 0.16 and 1.84 ± 0.20 in the group of patients with type 2 diabetes compared to 1.12 ± 0.12; 1.15 ± 0.11; 1.71 ± 0.15 and 2.69 ± 0.23 in the healthy control group (p = 0.002). Also, the insulin-stimulated serotonin response was significantly lower in patients with type 2 diabetes (Fig. 2): The percentage increase in blood-flow after co-infusion of insulin compared to serotonin alone was 45.96 ± 11.56%; 67.40 ± 18.11% and 84.57 ± 25.73% in the healthy control group and 26.48 ± 7.74%; 26.40 ± 11.52% and 19.75 ± 13.87% (p = 0.02) in the group of patients with type 2 diabetes.

**Figure 3:**
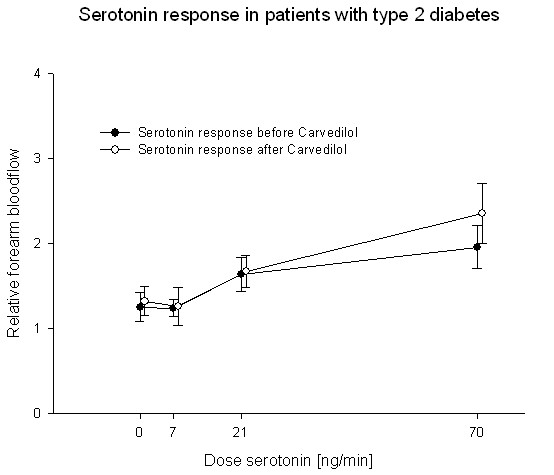
**Forearm blood-flow was not changed in the group of patients with type 2 diabetes after treatment with carvedilol (white circle) compared to the response before treatment (black circle)**.

**Figure 4:**
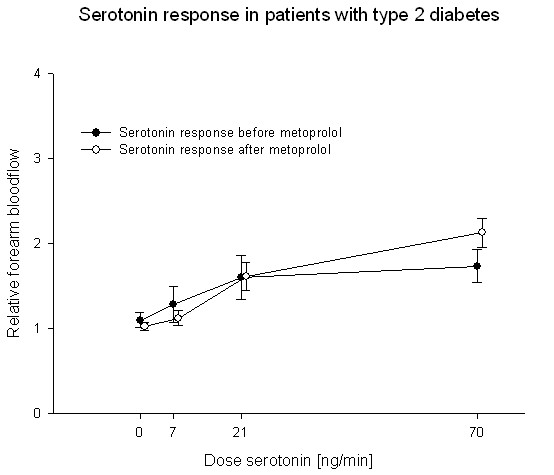
**Forearm blood-flow was not changed in the group of patients with type 2 diabetes after treatment with metoprolol (white circle) compared to the response before treatment (black circle)**.

Treatment with carvedilol or metoprolol did not change endothelium-dependent vasodilation. Before treatment the relative blood-flow was 1.25 ± 0.14; 1.24 ± 0.08; 1.64 ± 0.16 and 1.96 ± 0.14 in the carvedilol group at serotonin doses of 0, 7, 21 and 70 ng/minute respectively. After two months treatment with carvedilol, the relative blood-flow was 1.32 ± 0.14; 1.26 ± 0.18; 1.67 ± 0.15 and 2.35 ± 0.29 (P = 0.22) (Fig. 3). In the metoprolol group relative blood-flow was 1.10 ± 0.08; 1.28 ± 0.21; 1.60 ± 0.26 and 1.73 ± 0.20 before treatment and 1.03 ± 0.05; 1.12 ± 0.09; 1.62 ± 0.17 and 2.13 ± 0.17 after two months treatment (p = 0.30) (Fig. 4).

**Figure 5:**
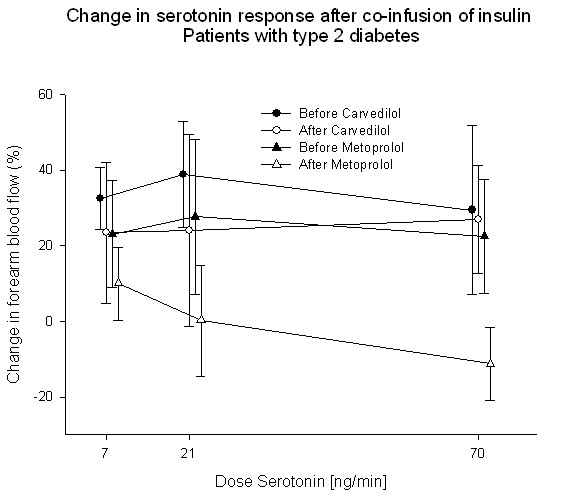
**The percentage increase in forearm blood-flow after co-infusion of serotonin and insulin was decreased after treatment with metoprolol (white triangle) compared to the blood-flow before this treatment (black triangle)**. The increase in forearm blood-flow was not changed by treatment with carvedilol.

After two months treatment with carvedilol the percentage increase in blood-flow after co-infusion with insulin was unchanged whereas treatment with metoprolol deteriorated the insulin-stimulated response significantly (Fig. 5). The percentage change after co-infusion of insulin in the group of patients treated with metoprolol was 29.77% ± 29.83; 31.44% ± 31.01 and 60.19% ± 17.89 before treatment and 41.46% ± 20.09; -9.45% ± 14.64 and -33.80% ± 23.38 after treatment (p = 0.007) at the three dose levels of serotonin respectively.

**Figure 6:**
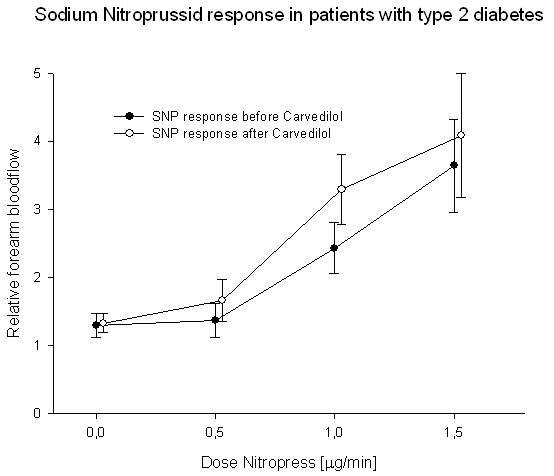
**Forearm blood-flow after stimulation with sodium nitroprusside was unchanged after treatment with carvedilol**.

**Figure 7:**
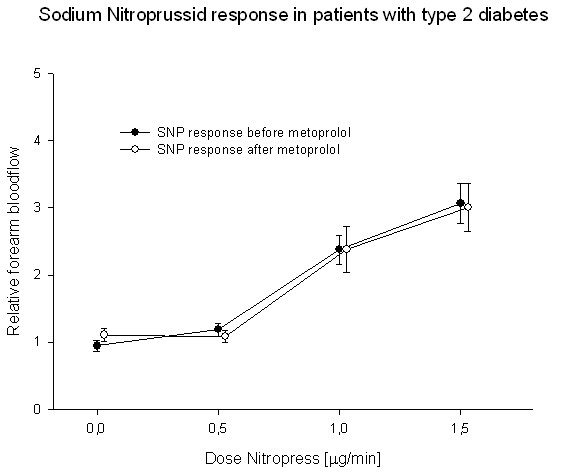
**Forearm blood-flow after stimulation with sodium nitroprusside was unchanged after treatment with metoprolol**.

Endothelium independent vasodilation, assessed after infusions of sodium nitroprusside, was unchanged after treatment with either of the two beta blockers (Fig 6 and 7).

L-NMMA co-infusion abolished the increase in blood-flow during serotonin and insulin co-infusion in both the carvedilol and metoprolol group at baseline and was not changed by either treatment with carvedilol or metoprolol.

## Discussion

The main result of this study is that insulin-stimulated endothelial function remained preserved during treatment with carvedilol and blunted during treatment with metoprolol, whereas endothelium-dependent and endothelium-independent vasodilation was unchanged in both groups.

The lack of effect of carvedilol in endothelial function in the absence of stimulation with insulin, is somehow in contrast with a recent study where carvedilol has shown to improve endothelial function assessed by measures on albuminuria and measures on brachial reactivity by ultrasound, compared with metoprolol [14]. It cannot be ruled out that we could have demonstrated an improvement of the serotonin-stimulated flow in a larger population but intra-arterial co-infusion of insulin and serotonin provides a unique possibility to assess specifically insulin sensitivity of the endothelium [15], whereby it further supports that an improved vascular nitric oxide reactivity is the main mechanism that accounts for the beneficial effects of carvedilol. This is additionally supported by the observation in our study that co-infusion of serotonin, insulin and L-NMMA totally abolished vasodilation both before and after treatment with either of the two beta blockers. These findings could not be explained by changes in either blood pressure or metabolic glucose control. Furthermore, it is possible that a lower CRP measured at baseline in the carvedilol group accounts for the lack of potential to improve serotonin response (Table 1). Nevertheless, this difference at baseline rather strengthens our study, since in spite of a "healthier" condition of the carvedilol group it was possible to improve the insulin-stimulated serotonin response. Insulin resistance is an independent risk factor of developing cardiovascular disease [16]. Diabetes is a condition with insulin resistance including vascular insulin resistance [8]. This was also found in our study. By improving metabolic glucose control in patients with type 2 diabetes, vascular insulin resistance also improves [8]. Insulin stimulated vasodilation has been found to be NO dependent [17]. A blunted insulin stimulated vasodilation itself leads to vasoconstriction and is thereby proatherogenic. Co-morbidity with hypertension, ischemic heart disease or heart failure is common in patients with type 2 diabetes. Treatment with beta adrenergic blockers is therefore often necessary to reduce their total risk of cardiovascular disease. As insulin resistance serves as a key role between diabetes and cardiovascular disease, it is of importance that beta blocker treatment does not aggravate insulin resistance. Vascular insulin resistance deteriorates glucose supply and thereby utilization in peripheral tissue. Vascular insulin resistance may therefore be an important factor when treating patients with type 2 diabetes or metabolic syndrome. The importance of the findings in this study for the prognosis of patients is unknown, but the result could inspire to further studies of the importance of vascular insulin sensitivity given the favorable effects of carvedilol compared to metoprolol observed in the COMET study (The Carvedilol or Metoprolol European Trial) [3,4].

Carvedilol has been found superior to metoprolol in the control of glucose metabolism in patients with type 2 diabetes and hypertension [2]. Also studies show that carvedilol does not deteriorate insulin resistance, as it was found in a direct comparison with metoprolol [18]. In the study by Jacob et al, insulin sensitivity was measured by use of the euglycemic hyperinsulinaemic clamp method and the study included patients with hypertension, but not diabetes. Compared to atenolol, a selective beta-1 adrenergic receptor blocker, carvedilol has also shown a more favorable effect on systemic glucose metabolism [19], whereas atenolol and metoprolol both decreased insulin sensitivity to a similar level, when compared directly with an euglycemic hyperinsulinaemic clamp [20]. These studies all show a systemic change in insulin resistance, whereas our study shows that the two beta-blockers, metoprolol and carvedilol, have a differential effect on vascular insulin sensitivity, with an advantage in favor of carvedilol.

In the group of patients treated with metoprolol we found an increase in body weight of 1.8 kg (p = 0.02) after 2 months of treatment. In contrast to this, no significant weight gain was found in the group of patients treated with carvedilol. This is in accordance with the weight gain seen after treatment with beta-blockers in large clinical trials [21]. In the GEMINI trial, a significant weight gain of 1.2 kg was found in the metoprolol arm compared to a non-significant weight gain in the carvedilol arm [2]; whether this can explain the general metabolic disadvantages seen with metoprolol in large clinical trials is uncertain. Whether the weight gain in the metoprolol group seen in our study is associated with deterioration of insulin-stimulated endothelial function is also not known. An inverse association between weight and adiponectin level has been found [22] and low plasma-adiponectin levels are considered to be a predictor of cardiovascular disease in patients with type 2 diabetes [23]. In a recent study, metoprolol was found to decrease adiponectin level in hypertensive patients [24]. Adiponectin could play a role in the relationship between vascular insulin resistance and treatment with metoprolol found in our study, and the weight-gain seen could also be an important factor.

The beta-1 adrenergic receptor blocker atenolol causes enhancement of endothelium-dependent vasodilation during short time infusion and during 3 months treatment [25,26]. In contrast, the non-selective beta-blocker propranolol causes attenuation of endothelial function during direct intra-arterial infusion and causes coronary artery vasoconstriction [25,27,28]. Four months treatment with carvedilol in patients with coronary artery disease improved endothelial function whereas no change was seen after short term treatment of 2 hours [29]. The long term effect on endothelial reactivity therefore seems to be dependent on the properties of the beta-blockers.

Carvedilol has additional adrenergic receptor blocking properties, beta-1, beta-2 and alfa-1, along with antioxidative properties [30] compared to metoprolol, a beta-blocker with beta-1 adrenergic receptor blocking properties. The effect of carvedilol on the endothelium may be explained by either increased endothelial NO-production or decreased NO-breakdown. Carvedilol stimulates endothelial nitric oxide production [31] and its hemodynamic effects are blunted during complete inhibition of NO-production [32]. In type 2 diabetes, an increased production of free radicals leads to an increased oxidative stress to the vascular wall [33]. Therefore carvedilol may have beneficial effects on endothelial dysfunction caused by oxidative stress in patients with type 2 diabetes.

Vascular studies of long-term treatment with carvedilol have shown to improve endothelial function in patients with coronary artery disease which was attributed to the antioxidative properties of carvedilol [34], but in a recent study improvement of endothelial function after treatment with carvedilol in patients with diabetes, no changes in markers of oxidative stress could be found [35].

As described, studies show inconclusive effects of the anti-oxidant property of carvedilol on endothelial function. We have not been able to demonstrate a benefit on endothelial function from treatment with carvedilol in patients with type 2 diabetes, despite the increased oxidative stress in this group of patients [36]. But our study indicates that carvedilol has supplemental effects and this might be of importance when treating diabetic vascular diseases; to our knowledge, this is the first study to demonstrate a direct effect on insulin-stimulated endothelial function in patients with type 2 diabetes, when treated with different generations of beta-blockers.

### Limitations to the study

The lack of changes in serotonin stimulated endothelial function after treatment with carvedilol or metoprolol might be caused by the small number of patients included in the study. Nevertheless we were able to demonstrate a difference between the insulin stimulated endothelial function in the group of patients treated with carvedilol and not even a trend of change in serotonin stimulated endothelial function. The number of patients included in the study might not be the only explanation to the lack of change after beta blocker treatment.

This a small interventional study with the purpose of finding changes in endothelial function between groups. Therefore changes in baseline characteristics could not be expected to be found with a statistical significance in this study. A large interventional study is needed to find the actual differences in endothelial function after treatment with either carvedilol or metoprolol with correction for the baseline characteristics and changes found in this study.

## Competing interests

The authors declare that they have no competing interests.

## Authors' contributions

BK carried out study design, examinations of the patients, data analysis and statistics and drafted the manuscript. TSH, CRM, LK, CTP and HD participated in designing the study, data analysis and statistics and critically revising the manuscript. AMP, BC, JRM and CRM participated in the design of the study and revising the manuscript.
